# Spinal Arteriovenous Malformation: Case Report and Review of the Literature

**DOI:** 10.7759/cureus.11614

**Published:** 2020-11-21

**Authors:** Tye Patchana, Paras Savla, Taha M Taka, Hammad Ghanchi, James Wiginton, Michael Schiraldi, Vladimir Cortez

**Affiliations:** 1 Neurosurgery, Riverside University Health System Medical Center, Moreno Valley, USA; 2 Neurosurgery, University of California, Riverside, USA; 3 Neurosurgery, Redlands Community Hospital, Redlands, USA; 4 Neurosurgery, Desert Regional Medical Center, Palm Springs, USA

**Keywords:** spinal arteriovenous malformation

## Abstract

Spinal arteriovenous malformations (AVMs) are a rare form of spinal blood vessel defect that results in vessel engorgement leading to clinical signs secondary to mass effect and ischemia. We present the patient’s clinical course following suspicion of spinal AVM along with a review of current classification and imaging modalities.

## Introduction

Spinal arteriovenous malformations (AVMs) are a rare and heterogeneous group of abnormally developed spinal blood vessels associated with an increased risk for hemorrhage and morbidity. Specifically, these malformations lead to shunting of blood from arteries to veins with an abnormal capillary bed. Annually, around 300 cases of spinal AVMs present in hospitals and require treatment [1]. Ultimately, this rarity and heterogeneity of AVMs lead to widely diverse treatment options which are dependent on the classification of AVM. For this reason, it is important to gain an understanding of the classification system of AVMs. While many classification systems have been used as further understanding of AVM development continues, our report will focus on the more widely used classification system of Anson and Spetzler in 1992 [2].

Pathophysiology

The mechanism of the development of spinal AVMs is not completely understood; however, the majority occur at birth rather than later in life [2]. Due to the shunting of arteriole blood to the venous system without capillary access and resistance, over 70% of arterial pressure is transmitted to the venous system [3]. Venous hypertension can precipitate many neurological deficits secondary to mass effect and normal spinal blood flow disruption along with increased risk for hemorrhage [4].

Classification

Type I: Spinal Dural Arteriovenous Fistula

Spinal dural arteriovenous fistulas (DAVFs) are the most common type of spinal vascular malformation, accounting for up to 85% of lesions [5]. Spinal DAVF commonly affects men, with a male:female ratio of 5:1. Additionally, this type of sAVM presents as slowly progressive leg weakness, back pain, or radicular pain in patients between 50 and 60 years old [6]. As their name implies, DAVFs are fistulas existing on the dural surface. These fistulas drain intradural through the nidus, or the AV communication, in a retrograde manner through the medullary vein and towards the coronal venous plexus. Therefore, the likely cause of the clinical presentation is the engorgement of the venous plexus.

Type II: Intramedullary Arteriovenous Malformation

Intramedullary AVMs is a congenital malformation that most commonly occurs within the thoracolumbar region, specifically the T4 and L3 levels [7]. Clinically, type II AVMs commonly present in young adults with an average age within the mid-20s [8]. Patients can present with myelopathy secondary to mass effect, ischemia, or hemorrhage. A previous study with a pooled analysis of multiple studies type II AVM hemorrhage determined a 4% annual hemorrhage rate which increased to 10% for AVMs with previous hemorrhage [9]. The increased likelihood of hemorrhage with intramedullary AVMs contributes to its higher rate of mortality.

Type III: Extradural-Intradural Arteriovenous Malformations 

Intradural-extradural spinal AVMs, also referred to as Juvenile vascular malformations, is a rare, complex, and locally aggressive lesion which can occupy bone, muscle, dura, spinal cord, and nerve roots [10]. This lesion commonly presents within younger patients, leading to an average age of 15.0 years (SD of 10.5) in one study of 51 patients [11]. While type III AVMs can present similarly to type II, with acute hemorrhages and myelopathy, they can be differentiated with the presence of signs of local tissue involvement. One example is the presence of spinal instability due to vertebral body involvement and degeneration after AVM hemorrhage [12]. The likelihood of type III AVMs having multiple feeding vessels makes jeopardizes the patient’s surgical candidacy; therefore, the main method of treatment is embolization for symptomatic relief and hemorrhage protection [11].

Type IV: Intradural Perimedullary Arteriovenous Fistula

Intradural perimedullary AVFs are a rare fistula caused by the presence of a shunt between a radicular artery and intradural veins leading to engorgement of these veins. While these lesions are rare and the true incidence rate is difficult to define, previous studies ranged from 4% to 17% prevalence in a large series of spinal AVMs [13]. Clinically, these lesions present with progressive myelopathy, acute neurological deficits, or subarachnoid hemorrhage. Due to the possibility of SAH, embolization is normally used to eliminate the fistula [14].

Imaging/detection

Spinal Digital Subtraction Angiography

Spinal digital subtraction angiography (DSA) is the current gold standard in visualizing and characterizing spinal AVMs prior to treatment. Spinal DSA is often labeled as a risky procedure leading to many complications; however, a recent study by Chen and Gailloud demonstrated that this label is a cause of historical data where multiple intraoperative factors led to a higher complication rate. In reality, the study concluded a low risk of neurological and systemic complications associated with DSA [15]. It is important to note that recent studies have demonstrated operator-dependent and avoidable missed diagnoses in patients. These factors included: documented but not identified lesions, lack of documenting regions of interest, inadequate injection leading to poor visualization, and involvement of vessels outside of the spine [16].

MRI

MRI is the modality of choice for initial visualization for spinal AVMs. While MRI lacks spatial and temporal specificity in comparison to DSA, its use in assessing spinal cord and surrounding structures can help narrow the differential diagnosis. Important signs that can help direct surgeons to AVMs are the presence of cord edema with increase T2 signal and flow voids. Additionally, a dilated intervertebral vein can also be visualized on T2-weighted imaging and should point towards AVM diagnosis [17].

MRA

Magnetic resonance angiography (MRA) is often a supplement of MRI that serves to identify the number of possible arterial feeders that supply the malformation. Therefore, this modality serves a facilitatory role in further imaging the spinal AVM through DSA [18]. Additionally, the use of MRI can lead to significantly decreased levels of radiation and the volume of contrast in spinal angiography [18].

CTA

Computed tomographic angiography (CTA) has been proven as a viable option for spinal AVM visualization. Recent advancements and usage of multidetector spiral CTA have increased the spatial resolution allowing for increased usage of this modality [19]. However, the spatial resolution is superior in DSA, making it a more useful modality for pre-operative planning [19].

## Case presentation

A 22-year-old male presented with a chief complaint of progressive bilateral lower extremity weakness and numbness. The patient stated that he had gone to a different hospital after experiencing lower extremity numbness, where evaluations were performed and he was discharged. However, he was unable to walk home at this time. Due to the persistence of his symptoms, the patient presented to our institution for the reevaluation of his continued bilateral lower extremity plegia.

**Figure 1:**
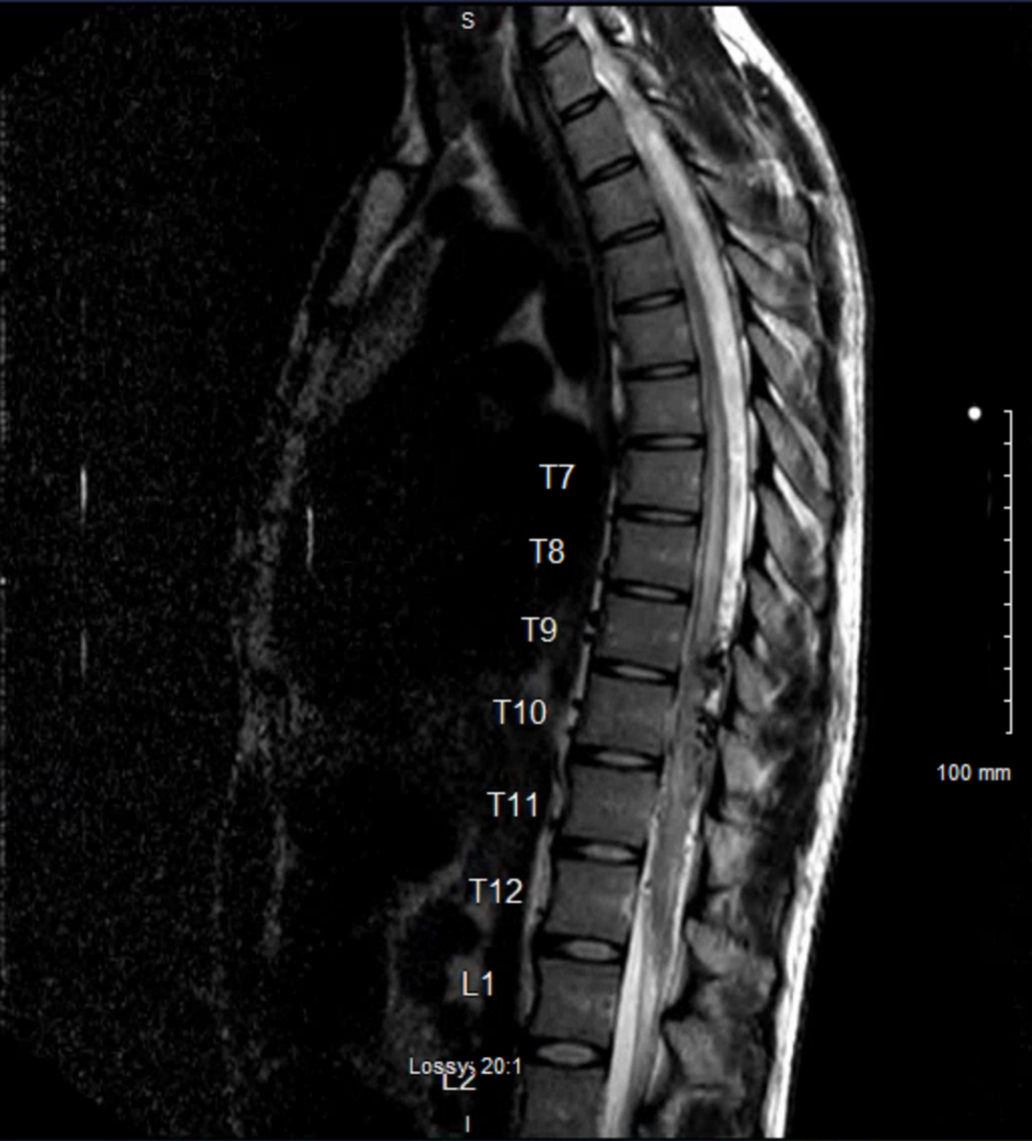
Preoperative MRI with and without contrast, T2 weighted.

On physical examination, the patient presented with saddle anesthesia. While the patient denied bowel or bladder incontinence, a Foley catheter was inserted upon admission for urinary retention. By American Spinal Cord Injury Association (ASIA) assessment, the patient was ASIA A with a T11 sensory level. A thoracic spine MRI with and without contrast was ordered, which raised suspicion for spinal AVM at T9-T10, as seen on T2 weighted imaging (Figure 1). Differential diagnoses included trauma, tumor, and other vascular abnormality.

**Figure 2:**
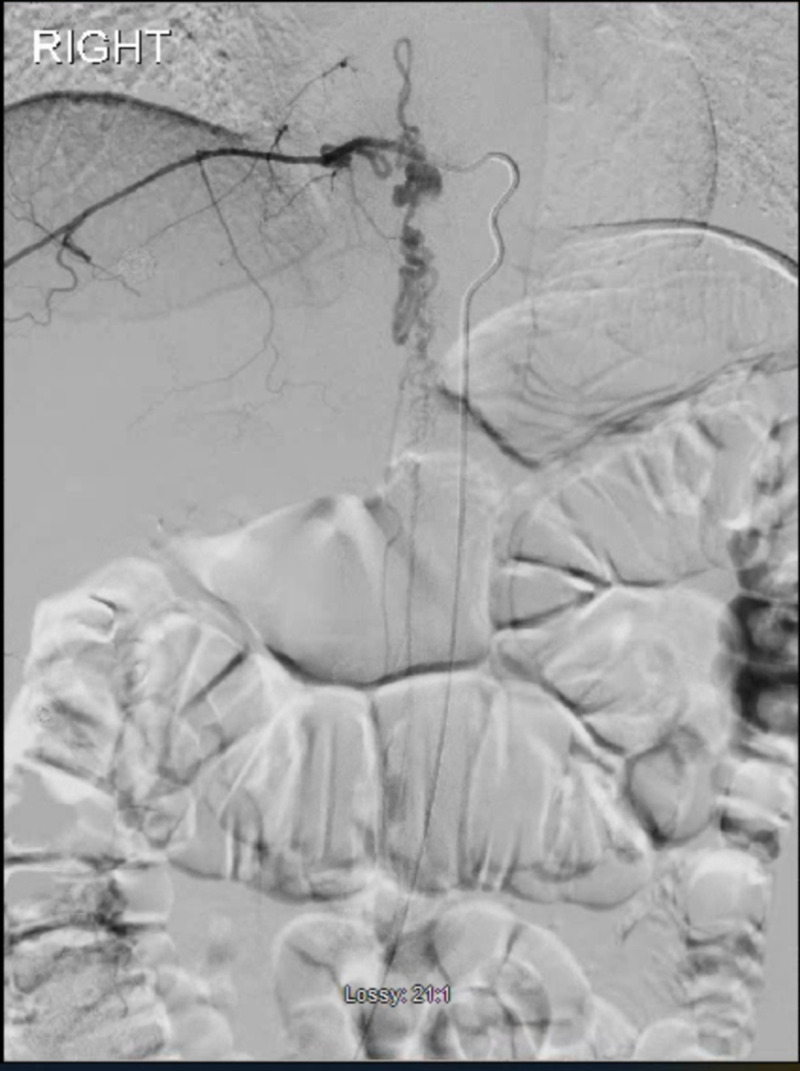
Spinal digital subtraction angiogram showing the AVM with the nidus at the right pedicle of T9. AVM: arteriovenous malformations

**Figure 3:**
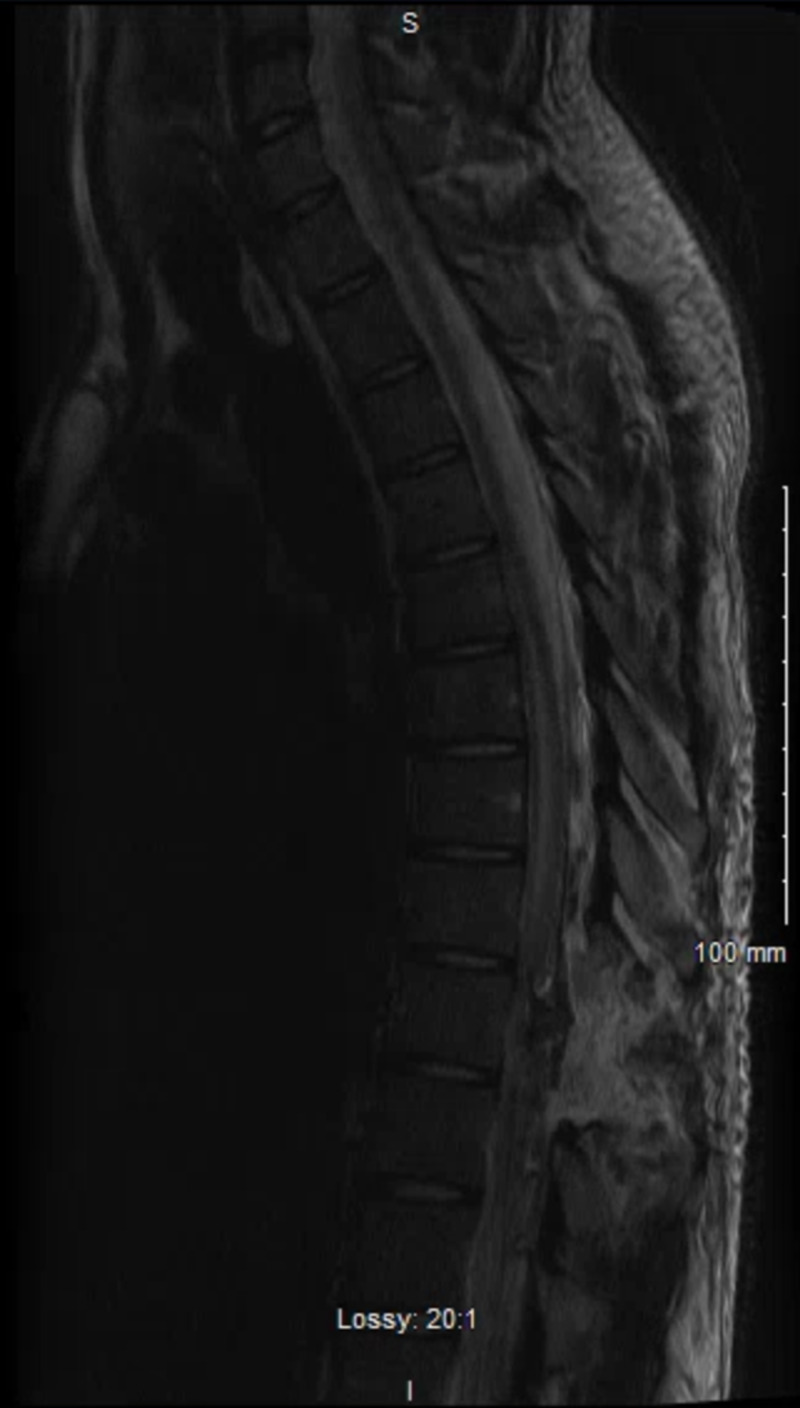
Postoperative MRI with and without contrast, T2 weighted.

The patient was taken for preoperative spinal digital subtraction angiogram for characterization of the lesion and possible embolization of the AVM. The nidus was found to be centered around the right pedicle of T9, about 1 cm in size (Figure 2). The lesion extended from T7 through T11. The patient was taken from the angiography suite to the operating room (OR) for surgery. Right-sided hemi-laminectomy and durotomy were performed at T8-T10 which revealed a feeding artery piercing through the dura near the inferior border of the right T9 pedicle surrounded by many arterialized veins. Multiple straight clips (five in total) were placed until no flow was detected within the vascular malformation on auscultation with micro-doppler. Postoperatively, the patient was taken back to the angiography suite to confirm the obliteration of the AVM. Digital subtraction angiography showed no residual AVM originating from the pedicle of T9. It also confirmed the preservation of the artery of Adamkiewicz. A post-operative MRI was also obtained to confirm the obliteration of the AVM (Figure 3).

Postoperatively, the patient remained an ASIA A with T11 sensory level, along with patchy but unreliable sensation to the bilateral lower extremities. The patient was discharged to acute inpatient rehabilitation with his Foley catheter in place due to ongoing retention and being bedbound. The patient was seen for his postoperative visit to the clinic, with a stable presentation. The patient continues to remain in acute inpatient rehabilitation and shows no signs or symptoms of AVM recurrence.

## Discussion

Spinal AVMs are a heterogeneous group of vascular lesions with a clinical presentation that is dependent on the affected spinal segment as well as the extensiveness of the malformation. We present a case of a 22-year-old African American man presenting with saddle anesthesia and progressive bilateral lower extremity numbness and weakness secondary to spinal AVM. Based on the imaging characters, intraoperative findings, and patient demographics, this was likely a type III AVM. Although rare, our patient fell into the classic presentation and age group for this type of AVM. It is also notable that although these AVMs typically are embolized for symptomatic relief, in this case, surgical clipping was the more amenable procedure. The patient tolerated the procedure well and has had improvement in his back pain. While his ASIA level has not improved, he does report subjective patchiness of sensation in his bilateral lower extremities.

Early suspicion, workup, diagnosis, and management could have resulted in the improved neurological outcome in this case. As with other compressive spinal cord pathology, such as a tumor, progressive deficits can become permanent [11]. Any further delay in the treatment of this patient may have led to further ascending deficits and possibly death from the expanding AVM and possible extensive rupture [10]. Additionally, while the risk of recurrence in adult AVM patients is low, a long-term follow-up to monitor recurrence is important. This is especially true in patients under the age of 18 as previous case reports/series cited a recurrence rate between 5.5% and 17.5% with the recurrence time ranging from one to five years after complete resection [20].

A key factor to be noted at the level of this lesion is the proximity of the artery of Adamkiewicz, which typically runs on the left side of the aorta from T8-L2. Injury to this artery can cause severe spinal cord ischemia similar to anterior spinal artery syndrome, resulting in neurological deficits including urinary/fecal incontinence and lower extremity weakness and paresis [12,15]. While our patient already had a complete motor loss in his bilateral lower extremities, he did have the potential to recover bowel and bladder function; thus, preservation of this artery was of vital importance. In fact, the location of this artery in relation to our patient’s AVM contributed to the necessity for open surgery rather than embolization.

## Conclusions

We present a case of a 22-year-old African American man with a progressive bilateral lower extremity numbness and weakness prior to the diagnosis of spinal AVM. Subsequently, imaging demonstrated a T8-T10 spinal AVM which was treated with occlusion of the feeding vessels through vascular clips. This case highlights the clinical progression and treatment of spinal AVMs along with a showcasing of a classification system and current imaging modalities.
